# Supplementary material for: Psychological consequences of COVID-19 home confinement: The ECLB-COVID19 multicenter study
Source: PLoS One. 2020 Nov 5;15(11):e0240204. doi: 10.1371/journal.pone.0240204 (PMC7643949; doi:10.1371/journal.pone.0240204)
Supplement: S1 File — (PDF) [file pone.0240204.s001.pdf]

Effects of Home Confinement on Multiple Lifestyle Behaviours During the COVID-19 Outbreak (ECLB-COVID19)

In March 2020, the World Health Organization (WHO) made the assessment that COVID-19 can be characterized as a pandemic. WHO and public health authorities around the world are acting to contain the COVID-19 outbreak through home confinement. However, this time of crisis is generating stress throughout the population.
The present survey aims to assess, in many countries, the effects of home confinement on multiple lifestyle behaviours during the COVID-19 outbreak. Identifying the exact changes in lifestyle behaviours during the confinement period (compared to before confinement) may help to provide efficient and effective suggestions/solutions (e.g., ICT-based solutions) to mitigate the possible unwanted psychosocial effects of home confinement.
* Required

Data Privacy/Security

Data protection and privacy is of the utmost importance. The present anonymous online survey was designed only for research purpose. In our research project we give special care to data privacy and security and strives to protect the collected data against any unauthorized access by third parties with taking into consideration the latest regulations in terms of data protection.
Accordingly, Information collected in the ECLB-COVID19 is kept in strictest confidence. During the informed consent process, survey participants are assured that data collected will be used only for research purposes and will not be disclosed or released to others without the consent of the individual. However, because we are using Google Forms, participants shall also acknowledge Google's privacy policy (https://policies.google.com/privacy?hl=en).
You must be 18 or older to participate in this study.

Consent of participation

Investigators declare that all data would be used only for research purposes (e.g., conferences and scientific publications) and not be disclosed or released to others without the consent of the individual. Participants' answers are anonymous and confidential according to Google's privacy policy (https://policies.google.com/privacy?hl=en). Participants don't have to mention their names or contact information. In addition, participant can stop participating in the study and can leave the questionnaire at any stage before the submission process and their responses will not be saved. Response will be saved only by clicking on "submit" button. By completing this survey, you are acknowledging the above approval form and you are consenting to voluntarily participate in this anonymous study. Please be honest in your responses.

If you are using your smartphone to answer this questionnaire, please drag the cursor to see all the possible answers

1. Country (first letter in uppercase) *

____

2. City *

3. Gender *

Mark only one oval.

- Male
- Female
- Other:

4. Age (years old) *

5. Level of Education *

Mark only one oval.

- No schooling completed
- High school graduate, diploma or the equivalent
- Professional degree
- Bachelor’s degree
- Master/doctorate degree

6. Marital status *

Mark only one oval.

- Single
- Married/Living as couple
- Widowed/Divorced/Separated

7. What is your current employment status? *

Check all that apply.

- [ ] Employed for wages
- [ ] Self-employed
- [ ] Out of work/Unemployed
- [ ] A student
- [ ] Retired
- [ ] Unable to work
- [ ] Problem caused by COVID-19 (e.g., leaving work, lower salary etc.)

Other: [ ] ____________________

8. What is your current state of health? *

Check all that apply.

- [ ] Healthy
- [ ] With risk factors for cardiovascular disease (e.g., high blood pressure, smoking, diabetes mellitus, obesity, high blood cholesterol, sedentarity, etc.)
- [ ] With cardiovascular disease (e.g., atherosclerosis, stroke, heart failure, heart disease, etc.)
- [ ] With cognitive impairment

9. Including yourself, how many people live in your household? *

Mark only one oval per row.

	1	2	3	4	5	more than 5
Before confinement	( )	( )	( )	( )	( )	( )
During confinement	( )	( )	( )	( )	( )	( )

10. Did you think that you are in need of psychosocial support ? *

Mark only one oval per row.

	Never	Rarely	Sometimes	Often	All times
Before confinement	( )	( )	( )	( )	( )
During confinement	( )	( )	( )	( )	( )

Short Life Satisfaction and Mental Well-being Questionnaires

Short Life Satisfaction questionnaire

11. 1. In most ways my life is close to my ideal. *

Mark only one oval per row.

	Strongly disagree	Disagree	Slightly disagree	Neither Agree Nor disagree	Slightly agree	Agree	Strongly Agree
Before confinement	⬭	⬭	⬭	⬭	⬭	⬭	⬭
During confinement	⬭	⬭	⬭	⬭	⬭	⬭	⬭

12. 2. So far I have gotten the important things I want in life *

Mark only one oval per row.

	Strongly disagree	Disagree	Slightly disagree	Neither Agree Nor disagree	Slightly agree	Agree	Strongly Agree
Before confinement	⬭	⬭	⬭	⬭	⬭	⬭	⬭
During confinement	⬭	⬭	⬭	⬭	⬭	⬭	⬭

13. 3. I am satisfied with my life. *

Mark only one oval per row.

	Strongly disagree	Disagree	Slightly disagree	Neither Agree Nor disagree	Slightly agree	Agree	Strongly Agree
Before confinement	⬭	⬭	⬭	⬭	⬭	⬭	⬭
During confinement	⬭	⬭	⬭	⬭	⬭	⬭	⬭

The Short Warwick-Edinburgh Mental Well-being Scale (SWEMWBS)

14. 1. I've been feeling optimistic about the future *

Mark only one oval per row.

	Never	Rarely	Sometimes	Often	All times
Before confinement	⬭	⬭	⬭	⬭	⬭
During confinement	⬭	⬭	⬭	⬭	⬭

15. 2. I've been feeling useful *

Mark only one oval per row.

	Never	Rarely	Sometimes	Often	All times
Before confinement	⬭	⬭	⬭	⬭	⬭
During confinement	⬭	⬭	⬭	⬭	⬭

16. 3. I’ve been feeling relaxed *

Mark only one oval per row.

	Never	Rarely	Sometimes	Often	All times
Before confinement					
During confinement					

17. 4. I’ve been dealing with Problems well *

Mark only one oval per row.

	Never	Rarely	Sometimes	Often	All times
Before confinement					
During confinement					

18. 5. I’ve been thinking clearly *

Mark only one oval per row.

	Never	Rarely	Sometimes	Often	All times
Before confinement					
During confinement					

19. 6. I’ve been feeling close to other people *

Mark only one oval per row.

	Never	Rarely	Sometimes	Often	All times
Before confinement					
During confinement					

20. 7. I've been able to make up my own mind about things *

Mark only one oval per row.

	Never	Rarely	Sometimes	Often	All times
Before confinement	⬭	⬭	⬭	⬭	⬭
During confinement	⬭	⬭	⬭	⬭	⬭

Social Participation Questionnary (SPQ)

21. 1. Visited family/family visit *

Mark only one oval per row.

	Never	Rarely	Sometimes	Often	All times
Before confinement	⬭	⬭	⬭	⬭	⬭
During confinement	⬭	⬭	⬭	⬭	⬭

22. 2. Visited friends or neighbours/friends or neighbours visit *

Mark only one oval per row.

	Never	Rarely	Sometimes	Often	All times
Before confinement	⬭	⬭	⬭	⬭	⬭
During confinement	⬭	⬭	⬭	⬭	⬭

23. 3. Attended church or a religious activity/group *

Mark only one oval per row.

	Never	Rarely	Sometimes	Often	All times
Before confinement	⬭	⬭	⬭	⬭	⬭
During confinement	⬭	⬭	⬭	⬭	⬭

24. 4. Used the internet/social media for communication *

Mark only one oval per row.

	Never	Rarely	Sometimes	Often	All times
Before confinement					
During confinement					

25. 5. Phone call for social communication *

Mark only one oval per row.

	Never	Rarely	Sometimes	Often	All times
Before confinement					
During confinement					

26. 6. Gone to a café/restaurant, bar or party *

Mark only one oval per row.

	Never	Rarely	Sometimes	Often	All times
Before confinement					
During confinement					

27. 7. Gone to the cinema or theatre or sport event *

Mark only one oval per row.

	Never	Rarely	Sometimes	Often	All times
Before confinement					
During confinement					

28. 8. Gone to the gym or exercise class *

Mark only one oval per row.

	Never	Rarely	Sometimes	Often	All times
Before confinement					
During confinement					

29. 9. Gone to a class *

Mark only one oval per row.

	Never	Rarely	Sometimes	Often	All times
Before confinement					
During confinement					

30. 10. Had social contact through other activities *

Mark only one oval per row.

	Never	Rarely	Sometimes	Often	All times
Before confinement					
During confinement					

31. 11. School-related group *

Mark only one oval per row.

	No	Yes
Before confinement		
During confinement		

32. 12. Volunteer organization or group *

Mark only one oval per row.

	No	Yes
Before confinement	⬭	⬭
During confinement	⬭	⬭

33. 13. Ethnic group *

Mark only one oval per row.

	No	Yes
Before confinement	⬭	⬭
During confinement	⬭	⬭

34. 14. Other group (e.g., sport, service, etc.) *

Mark only one oval per row.

	No	Yes
Before confinement	⬭	⬭
During confinement	⬭	⬭

Short Mood and Feelings Questionnaire (SMFQ)

35. 1. I felt miserable or unhappy *

Mark only one oval per row.

	0 (not true)	1 (sometimes)	2 (true)
Before confinement	⬭	⬭	⬭
During confinement	⬭	⬭	⬭

36. 2. I didn't enjoy anything at all *

Mark only one oval per row.

	0 (not true)	1 (sometimes)	2 (true)
Before confinement			
During confinement			

37. 3. I felt so tired I just sat around and did nothing *

Mark only one oval per row.

	0 (not true)	1 (sometimes)	2 (true)
Before confinement			
During confinement			

38. 4. I was very restless *

Mark only one oval per row.

	0 (not true)	1 (sometimes)	2 (true)
Before confinement			
During confinement			

39. 5. I felt I was no good anymore *

Mark only one oval per row.

	0 (not true)	1 (sometimes)	2 (true)
Before confinement			
During confinement			

40. 6. I cried a lot *

Mark only one oval per row.

	0 (not true)	1 (sometimes)	2 (true)
Before confinement			
During confinement			

41. 7. I found it hard to think properly or concentrate *

Mark only one oval per row.

	0 (not true)	1 (sometimes)	2 (true)
Before confinement			
During confinement			

42. 8. I hated myself *

Mark only one oval per row.

	0 (not true)	1 (sometimes)	2 (true)
Before confinement			
During confinement			

43. 9. I was a bad person *

Mark only one oval per row.

	0 (not true)	1 (sometimes)	2 (true)
Before confinement			
During confinement			

44. 10. I felt lonely *

Mark only one oval per row.

	0 (not true)	1 (sometimes)	2 (true)
Before confinement			
During confinement			

45. 11. I thought nobody really loved me *

Mark only one oval per row.

	0 (not true)	1 (sometimes)	2 (true)
Before confinement			
During confinement			

46. 12. I thought I could never be as good as other people *

Mark only one oval per row.

	0 (not true)	1 (sometimes)	2 (true)
Before confinement			
During confinement			

47. 13. I did everything wrong *

Mark only one oval per row.

	0 (not true)	1 (sometimes)	2 (true)
Before confinement			
During confinement			

Physical Activity and Diet Behaviours Questionnaires

International Physical Activity Questionnaire - Short Form

48. 1. During a week, how many days did you do vigorous (hard) physical activities (e.g., lifting, digging, aerobics, or fast biking)? *

Mark only one oval per row.

	0 (Skip to question 3)	1	2	3	4	5	6	7
Before confinement	◯	◯	◯	◯	◯	◯	◯	◯
During confinement	◯	◯	◯	◯	◯	◯	◯	◯

49. 2. How much time (Minute/day) did you usually spend doing vigorous physical activities on one of those days?

(.....min) Before confinement AND (.....min) During confinement

____

50. 3. During a week, how many days did you do moderate physical activities (e.g., light loads, bicycling at a regular pace, or doubles tennis)? Do not include walking *

Mark only one oval per row.

	0 (Skip to question 5)	1	2	3	4	5	6	7
Before confinement	◯	◯	◯	◯	◯	◯	◯	◯
During confinement	◯	◯	◯	◯	◯	◯	◯	◯

51. 4. How much time did you usually spend doing moderate physical activities on one of those days?

(.....min) Before confinement AND (.....min) During confinement

____

52. 5. During a week, how many days did you do walk/recreation activity for at least 10 minutes at a time? *

Mark only one oval per row.

	0 (Skip to question 7)	1	2	3	4	5	6	7
Before confinement	⬭	⬭	⬭	⬭	⬭	⬭	⬭	⬭
During confinement	⬭	⬭	⬭	⬭	⬭	⬭	⬭	⬭

53. 6. How much time did you usually spend walking on one of those days?
(.....min) Before confinement AND (.....min) During confinement

____

54. 7. How much time did you spend sitting on a week day? *
(.....hours) Before confinement AND (.....hours) During confinement

____

55. 8. How likely are you to use internet, social media, apps , smart watch/phone, fitness tracker for physical activity purpose *

Mark only one oval per row.

	Never	Sometimes	Most of the time	Always
Before confinement	⬭	⬭	⬭	⬭
During confinement	⬭	⬭	⬭	⬭

Short Diet Behaviours Questionnaire

56. 1. How likely are you to have an unhealthy diet/food ?(high in calories from sugar or fat, colorants, salt and tropical oils; and low in fibre and vitamins (e.g., fried potato crisps/chips, cakes, white sauces) *

Mark only one oval per row.

	Never	Sometimes	Most of the time	Always
Before confinement	◯	◯	◯	◯
During confinement	◯	◯	◯	◯

57. 2. How often have you found yourself being eating out of control (binge-eating)? *

Mark only one oval per row.

	Never	Sometimes	Most of the time	Always
Before confinement	◯	◯	◯	◯
During confinement	◯	◯	◯	◯

58. 3. How many main meals do you eat a day (i.e., breakfast, lunch, dinner)? *

Mark only one oval per row.

	1-2	3	4	5	more than 5
Before confinement	◯	◯	◯	◯	◯
During confinement	◯	◯	◯	◯	◯

59. 4. How likely are you to have a snack between meals or a late night snack ? (Consider every fruit,every yoghurt, or a glass of milk etc. as a single snack) *

Mark only one oval per row.

	Never	Sometimes	Most of the time	Always
Before confinement	◯	◯	◯	◯
During confinement	◯	◯	◯	◯

60. 5. Do you engage in binge alcohol drinking? (5 or more drinks in a sitting) *

Mark only one oval per row.

	Never	Sometimes	Most of the time	Always
Before confinement	⬭	⬭	⬭	⬭
During confinement	⬭	⬭	⬭	⬭

61. 6. How likely are you to use internet, social media, apps for diet purpose (e.g., preparing meal, controling calories etc.) *

Mark only one oval per row.

	Never	Sometimes	Most of the time	Always
Before confinement	⬭	⬭	⬭	⬭
During confinement	⬭	⬭	⬭	⬭

Pittsburgh Sleep Quality Index (PSQI)

1. What time have you usually gone to bed at night?

62. 1.a. Before confinement (time to bed) *

____

Example: 8:30 AM

63. 1.b. During confinement (time to bed) *

____

Example: 8:30 AM

64. 2. How long (in minutes) has it usually taken you to fall asleep each night? *

(.....min) Before confinement AND (.....min) During confinement

____

3. What time have you usually gotten up in the morning?

65. 3.a. Before confinement (getten up time) *

Example: 8:30 AM

66. 3.b. During confinement (getten up time) *

Example: 8:30 AM

67. 4. How many hours of actual sleep did you get at night? (This may be different than the number of hours you spent in bed.) *

(.....hours) Before confinement AND (.....hours) During confinement

5. How often have you had trouble sleeping because you . . .

68. 5.a.Cannot get to sleep within 30 minutes *

Mark only one oval per row.

	No	Less than once a week	Once or Twice a week	Three or more times a week
Before confinement	⬭	⬭	⬭	⬭
During confinement	⬭	⬭	⬭	⬭

69. 5.b. Wake up in the middle of the night or early morning *

Mark only one oval per row.

	No	Less than once a week	Once or Twice a week	Three or more times a week
Before confinement	⬭	⬭	⬭	⬭
During confinement	⬭	⬭	⬭	⬭

70. 5.c. Have to get up to use the bathroom *

Mark only one oval per row.

	No	Less than once a week	Once or Twice a week	Three or more times a week
Before confinement	⬭	⬭	⬭	⬭
During confinement	⬭	⬭	⬭	⬭

71. 5.d. Cannot breathe comfortably, Cough or snoreloudly *

Mark only one oval per row.

	No	Less than once a week	Once or Twice a week	Three or more times a week
Before confinement	⬭	⬭	⬭	⬭
During confinement	⬭	⬭	⬭	⬭

72. 5.e. Feel too cold/hot *

Mark only one oval per row.

	No	Less than once a week	Once or Twice a week	Three or more times a week
Before confinement				
During confinement				

73. 5.f. Other reason (e.g., pain, use phone, bad dreams etc.) *

Mark only one oval per row.

	No	Less than once a week	Once or Twice a week	Three or more times a week
Before confinement				
During confinement				

74. 6. How often have you taken medicine to help you sleep (prescribed or "over the counter")? *

Mark only one oval per row.

	NO	Less than once a week	Once or Twice a week	Three or more times a week
Before confinement				
During confinement				

75. 7. How often have you had trouble staying awake while driving, eating meals, or engaging in social activity? *

Mark only one oval per row.

	NO	Less than once a week	Once or Twice a week	Three or more times a week
Before confinement	⬭	⬭	⬭	⬭
During confinement	⬭	⬭	⬭	⬭

76. 8. How much of a problem has it been for you to keep up enough enthusiasm to getthings done? *

Mark only one oval per row.

	No problem at all	Only a very slight problem	Somewhat of a problem	A very big problem
Before confinement	⬭	⬭	⬭	⬭
During confinement	⬭	⬭	⬭	⬭

77. 9. How would you rate your sleep quality overall? *

Mark only one oval per row.

	Very good	Fairly good	Fairly bad	Very bad
Before confinement	⬭	⬭	⬭	⬭
During confinement	⬭	⬭	⬭	⬭

This content is neither created nor endorsed by Google.

Google Forms
