# Supplementary material for: Psychological consequences of COVID-19 home confinement: The ECLB-COVID19 multicenter study
Source: PLoS One. 2020 Nov 5;15(11):e0240204. doi: 10.1371/journal.pone.0240204 (PMC7643949; doi:10.1371/journal.pone.0240204)
Supplement: S1 Table — (PDF) [file pone.0240204.s002.pdf]

S1 Table. Distribution of responses (%) in each item of the mental wellbeing questionnaire.

Question/responses			Mean ±SD	% of responses (Q1-Q7)					% of responses (total score)		
Num	Text	Time		Never	Rarely	Sometimes	Often	All times	Low	Medium	High
Q1	I've been feeling optimistic about the future	Before	4.08±0.91	1.7%	3.6%	17.0%	40.7%	37.0%	N/A		
		During	3.54±1.11	4.9%	11.1%	32.7%	28.3%	23.1%			
Q2	I've been feeling useful	Before	4.05±0.89	1.7%	3.2%	17.5%	44.0%	33.6%			
		During	3.62±1.13	5.4%	10.7%	26.1%	32.5%	25.3%			
Q3	I've been feeling relaxed	Before	3.38±0.94	3.4%	11.5%	38.9%	35.7%	10.5%			
		During	3.25±1.07	6.5%	16.1%	35.2%	29.9%	12.2%			
Q4	I've been dealing with Problems well	Before	3.88±0.81	1.2%	3.4%	21.6%	53.5%	20.2%			
		During	3.62±0.93	2.3%	8.1%	31.1%	41.9%	16.5%			
Q5	I've been thinking clearly	Before	3.99±0.77	0.7%	3.1%	17.2%	55.0%	24.1%			
		During	3.71±0.94	2.1%	7.8%	26.7%	43.6%	19.8%			
Q6	I've been feeling close to other people	Before	3.88±0.92	1.6%	5.3%	23.3%	43.4%	26.5%			
		During	3.26±1.16	7.8%	17.7%	30.8%	27.7%	16.0%			
Q7	I've been able to make up my own mind about things	Before	4.04±0.83	1.1%	3.2%	15.9%	50.0%	29.9%			
		During	3.72±1	3.0%	8.1%	25.6%	40.1%	23.2%			
Total Score		Before	27.3±4.37	N/A					5.6%	43.1%	51.4%
		During	24.73±5.18						18.5%	51.4%	30.2%
