# Supplementary material for: Psychological consequences of COVID-19 home confinement: The ECLB-COVID19 multicenter study
Source: PLoS One. 2020 Nov 5;15(11):e0240204. doi: 10.1371/journal.pone.0240204 (PMC7643949; doi:10.1371/journal.pone.0240204)
Supplement: S2 Table — (PDF) [file pone.0240204.s003.pdf]

S2 Table. Distribution of responses (%) in each item of the mood and feeling questionnaire.

Question/responses			Mean ± SD	% of responses (Q1-Q13)			% of responses (total score)	
Num	Text	Time		Not true	Sometimes	True	Presence of depression	Absence of depression
Q1	I felt miserable or unhappy	Before	0.49±0.57	55.0%	41.3%	3.7%	N/A	
		During	0.79±0.72	38.7%	44.1%	17.2%		
Q2	I didn't enjoy anything at all	Before	0.29±0.51	73.9%	23.5%	2.6%		
		During	0.6±0.7	52.8%	34.9%	12.3%		
Q3	I felt so tired I just sat around and did nothing	Before	0.46±0.6	59.8%	34.7%	5.5%		
		During	0.81±0.78	41.9%	35.5%	22.5%		
Q4	I was very restless	Before	0.46±0.6	59.9%	34.4%	5.7%		
		During	0.66±0.75	50.5%	33.0%	16.5%		
Q5	I felt I was no good anymore	Before	0.34±0.53	68.6%	28.7%	2.8%		
		During	0.55±0.71	57.2%	30.1%	12.7%		
Q6	I cried a lot	Before	0.39±0.6	67.2%	26.7%	6.0%		
		During	0.43±0.67	67.4%	22.4%	10.1%		
Q7	I found it hard to think properly or concentrate	Before	0.53±0.58	51.1%	44.4%	4.5%		
		During	0.77±0.74	40.9%	40.8%	18.3%		
Q8	I hated myself	Before	0.23±0.49	79.8%	17.0%	3.2%		
		During	0.32±0.6	75.1%	17.9%	7.1%		
Q9	I was a bad person	Before	0.15±0.39	85.6%	13.4%	1.1%		
		During	0.17±0.44	86.0%	11.3%	2.8%		
Q10	I felt lonely	Before	0.39±0.58	66.2%	29.0%	4.8%		
		During	0.59±0.73	55.6%	30.1%	14.3%		
Q11	I thought nobody really loved me	Before	0.26±0.52	77.6%	18.7%	3.7%		
		During	0.29±0.57	76.9%	17.4%	5.7%		
Q12	I thought I could never be as good as other people	Before	0.23±0.49	80.4%	16.4%	3.2%		
		During	0.26±0.54	78.6%	16.3%	5.1%		
Q13	I did everything wrong	Before	0.27±0.49	74.7%	23.3%	2.0%		
		During	0.27±0.49	75.1%	22.6%	2.3%		
Total Score		Before	4.49±4.41	N/A			7.4%	92.6%
		During	6.5±5.63				17.5%	82.5%
